# Phosphate Restriction Promotes Longevity via Activation of Autophagy and the Multivesicular Body Pathway

**DOI:** 10.3390/cells10113161

**Published:** 2021-11-13

**Authors:** Mahsa Ebrahimi, Lukas Habernig, Filomena Broeskamp, Andreas Aufschnaiter, Jutta Diessl, Isabel Atienza, Steffen Matz, Felix A. Ruiz, Sabrina Büttner

**Affiliations:** 1 Department of Molecular Biosciences, The Wenner-Gren Institute, Stockholm University, 106 91 Stockholm, Sweden; mahsa.ebrahimi@su.se (M.E.); Lukas.habernig@su.se (L.H.); filomena.broeskamp@su.se (F.B.); jutta.diessl@su.se (J.D.); steffenmatz@gmx.de (S.M.); 2 Department of Biochemistry and Biophysics, Stockholm University, 106 91 Stockholm, Sweden; andreas.aufschnaiter@dbb.su.se; 3 Instituto de Investigación e Innovación Biomédica de Cádiz (INIBICA), University of Cadiz, 11001 Cadiz, Spain; isabel.atienza@uca.es (I.A.); felix.ruiz@gm.uca.es (F.A.R.); 4 Institute of Molecular Biosciences, University of Graz, 8010 Graz, Austria

**Keywords:** lifespan, nutrient limitation, yeast, autophagy, Pho85, aging, polyphosphate, vacuole fusion, quiescence

## Abstract

Nutrient limitation results in an activation of autophagy in organisms ranging from yeast, nematodes and flies to mammals. Several evolutionary conserved nutrient-sensing kinases are critical for efficient adaptation of yeast cells to glucose, nitrogen or phosphate depletion, subsequent cell-cycle exit and the regulation of autophagy. Here, we demonstrate that phosphate restriction results in a prominent extension of yeast lifespan that requires the coordinated activity of autophagy and the multivesicular body pathway, enabling efficient turnover of cytoplasmic and plasma membrane cargo. While the multivesicular body pathway was essential during the early days of aging, autophagy contributed to long-term survival at later days. The cyclin-dependent kinase Pho85 was critical for phosphate restriction-induced autophagy and full lifespan extension. In contrast, when cell-cycle exit was triggered by exhaustion of glucose instead of phosphate, Pho85 and its cyclin, Pho80, functioned as negative regulators of autophagy and lifespan. The storage of phosphate in form of polyphosphate was completely dispensable to in sustaining viability under phosphate restriction. Collectively, our results identify the multifunctional, nutrient-sensing kinase Pho85 as critical modulator of longevity that differentially coordinates the autophagic response to distinct kinds of starvation.

## 1. Introduction

Cellular aging refers to the progressive functional decline of diverse and intertwined subcellular processes, resulting in decreased fitness and eventually cell death. Several of the molecular pathways implicated in the aging process are conserved across species, and central nutrient-sensing and -signaling pathways govern the lifespan in organisms ranging from yeast, nematodes and flies, to rodents and humans [1,2,3,4]. The downregulation of these nutrient-responsive pathways critically contributes to the beneficial effects of caloric restriction (the reduction of caloric intake without malnutrition), a regime that counteracts age-associated cellular dysfunction in most organisms tested, including yeast [5,6,7]. In response to starvation for glucose or other nutrients, yeast cells can enter quiescence, a non-proliferative state with rather heterogenous properties associated with high stress resistance and longevity [8]. While converging genetic programs and global responses to general nutrient deprivation are in place to adjust cell-cycle exit and stress responses, cells also possess genetic programs specifically tailored to the depletion of distinct macronutrients, such as carbon, nitrogen or phosphate, adapting cellular metabolism to the respective nutrient scarcity [9,10,11,12]. Moreover, the genes necessary to maintain viability during starvation differ substantially depending on the depleted nutrient [12]. Entry into stationary phase and quiescence due to exhaustion of glucose or nitrogen is associated with a severe remodeling of distinct organelles and subcellular structures and a prominent induction of autophagy, contributing to these adaptations [8]. Efficient upregulation of autophagy, the main bulk degradation and recycling pathway in eukaryotic cells, is essential to sustain survival upon glucose or nitrogen exhaustion, and genetic inactivation of autophagic flux causes premature death [13,14,15,16]. Besides the breakdown of cytoplasmic material via autophagy, the turnover of plasma membrane material via the multivesicular body (MVB) pathway has been suggested to contribute to cellular fitness during aging, in particular upon starvation for nitrogen [17,18,19]. While the cellular processes and signaling pathways involved in longevity upon glucose or nitrogen restriction are relatively well understood, not much is known about lifespan in phosphate-limited conditions. Phosphorus is an essential macronutrient required for a plethora of cellular processes. In all organisms, a relatively small amount of phosphorus is found in solution as inorganic phosphate (P_i_), while most of it exists as organic phosphate in phospholipids, phosphoproteins, DNA and RNA or is stored as polyphosphate (polyP), a linear polymer of P_i_ residues connected by high-energy phosphoanhydride bonds [20,21]. As cells encounter highly variable amounts of extracellular phosphate in nature, systems to cope with unpredictable oscillations of phosphate are essential to sustain viability. In yeast, the cyclin-dependent kinase Pho85 (the counterpart to mammalian kinase CDK5), its cyclin Pho80, its inhibitor Pho81 and the transcriptional activator Pho4 cooperate to adapt phosphate scavenging, storage and utilization to the cellular needs [22,23,24]. The short-term transcriptional changes upon acute phosphate starvation are well characterized [9,11,25,26,27,28,29]. However, not much is known about the processes and pathways governing long-term survival in phosphate-limited conditions, where cells sense a gradual decline of phosphate levels that drives entry into stationary phase.

Here, we show that phosphate exhaustion results in a prominent extension of lifespan that requires the coordinated activity of the MVB pathway and macroautophagy, the two main catabolic pathways delivering cargo to the vacuole for recycling. While the MVB pathway contributed to short-term survival upon phosphate restriction, autophagy was essential to sustain long-term viability. The stress response transcription factor Rim15, integrating signals from diverse nutrient-responsive kinases, was critical for longevity induced by phosphate restriction. Moreover, we demonstrate that Pho85 has opposing roles in glucose versus phosphate-depleted cells. Genetic inactivation of this kinase resulted in mild induction of autophagy and longevity upon glucose exhaustion. In contrast, induction of autophagy as well as full lifespan extension via phosphate restriction critically depended on the presence of the Pho85–Pho80 complex.

## 2. Materials and Methods

### 2.1. Yeast Strains and Growth Conditions

The *Saccharomyces cerevisiae* strains used in this study were created from the parental strain BY4742 (*MATα his3*∆1 *leu2*∆0 *lys2*∆0 *ura3*∆0) obtained from Euroscarf (distributed by Scientific Research and Development GmbH, Oberursel, Germany) and are listed in Supplemental Table S1. Gene disruption and chromosomal tagging were performed as described [30], and all oligonucleotides used for genomic modifications are listed in Supplemental Table S2. All strains were grown in synthetic complete (SC) medium, using 0.65% yeast nitrogen base (YNB) without phosphate (Formedium, CYN6703) and 2% glucose. After autoclavation, 30 mg/L of all amino acids (except 80 mg/L histidine, 200 mg/L leucine and 120 mg/L lysine), 30 mg/L adenine and 320 mg/L uracil were added. Phosphate was supplemented as NaH_2_PO_4_ to a final concentration of 0.2 mM in phosphate restriction (PR) or 7 mM in the control (Ctrl) medium, corresponding to the standard phosphate concentration in regular YNB. For caloric restriction (CR), SC medium with 0.17% YNB, containing 7 mM phosphate (BD), 0.5% (NH_4_)_2_SO_4_, all amino acid mixtures described above and 0.5% glucose were used. For nitrogen starvation (−N), SC medium with 0.17% YNB without amino acids and ammonium sulfate (BD) and 2% glucose was used. All cultures were grown at 28 °C and 145 rpm. Overnight cultures (ONCs) were grown in 3 mL SC medium supplemented with NaH_2_PO_4_ to a final concentration of 7 mM phosphate (Ctrl) for 16–18 h. ONCs were centrifuged at 3500 rpm for 3 min, the pellets were washed twice with phosphate-free SC medium and finally resuspended in 3 mL of phosphate-free SC medium. OD_600_ was determined and cells were used to inoculate 15-mL cultures of respective media, containing either 7 mM (Ctrl) or 0.2 mM (PR) phosphate, in baffled flasks to an OD_600_ of 0.1. Cultures were incubated at 28 °C and shaking at 145 rpm, and aliquots were taken at indicated time points during chronological aging. To test for any effects of media acidification, the pH of control cultures was adjusted to the pH of PR cultures using NaOH. For nitrogen starvation (−N) experiments, cells were inoculated in 20 mL Ctrl medium to OD_600_ of 0.2, grown for 8 h, pelleted, washed twice with water and diluted in 20 mL −N medium to OD_600_ of 2. For CR experiments, ONCs were grown in Ctrl medium before inoculation to OD_600_ of 0.1 in 15 mL of CR medium in baffled flasks. 

### 2.2. Growth Kinetics, Clonogenic Survival Plating and Spotting Assay

ONCs in Ctrl medium were washed in phosphate-free SC medium as described above, and cells were inoculated to an OD_600_ of 0.1 in media supplemented with NaH_2_PO_4_ to a final concentration of 0 mM, 0.1 mM, 0.2 mM, 0.5 mM, 1 mM or 7 mM in Honeycomb microplates^®^. OD_600_ was measured every 30 min for 24 h using a BioscreenC (Oy Growth Curves Ab Ltd., Helsinki, Finland). Samples were shaking at medium speed at 28 °C, which was stopped 5 s before each measurement. Data was acquired using the BioscreenC software ver. 1.0.0.87. For clonogenic survival plating, OD_600_ was measured at indicated days, aliquots were diluted in dH_2_O and 3.5 × 10^−5^ OD_600_ were plated on YPD agar plates. Colony-forming units (CFU) were quantified using a Scan300 (Interscience, Saint Nom la Brétèche, France) after 2 days of incubation at 30 °C. For serial dilution spotting assays, OD_600_ was measured at indicated days. Cultures were adjusted to OD_600_ of 0.2 in dH_2_O, serial diluted (1:5) and spotted on YPD agar plates using a Pin Replicator (V&P Scientific, Inc., San Diego, CA, USA). Pictures were taken after 1 day of incubation at 30 °C.

### 2.3. Determination of Survival via Flow Cytometry and Calculation of Median Lifespan

Cell death was determined using propidium iodide (PI) staining, indicative of plasma membrane rupture, as previously described [31]. At indicated time points during chronological aging, cells were harvested in 96-well plates by centrifugation at 4000 rpm for 5 min and stained with 250 μL PBS containing PI (Sigma-Aldrich 81845-100MG) with a final concentration of 500 μg/L, followed by incubation in the dark for 7 min. After centrifugation, cells were resuspended in PBS and PI positive and thus dead cells were quantified via flow cytometry using a BD LSRFortessa™ and the BD FACSDiva v8.0.1 software (BD Biosciences, San Jose, California, US) (30,000 cells were analyzed) or a Guava^®^ easyCyte HT with guavaSoft 3.3 software (Luminex Corporate, Austin, TX, USA) (5000 cells were analyzed). Median lifespan was defined as the time point at which 50% of cells in an individual culture were identified as PI-positive. To calculate the median lifespan, linear regression of PI positive cells between the last analyzed time point before reaching 50% and the first analyzed time point after reaching 50% PI positive cells was performed. Thereby, the median lifespan for each clone was calculated as [median lifespan = (50 intercept)/slope] and the average thereof was presented as the median lifespan of indicated strains.

### 2.4. Immunoblotting 

Six OD_600_ of cells were harvested at the indicated time points and proteins were extracted by chemical lysis using the TCA extraction method. The pellets were resuspended in 300 μL of lysis buffer (1.85 M NaOH, 7.5% μL β-mercaptoethanol) followed by incubation on ice for 10 min. Then, 300 μL of 55% TCA was added, samples were incubated for 10 min on ice, and centrifuged, at 10,000× *g* and 4 °C. TCA was removed completely and pellets were dissolved in urea loading buffer (200 mM Tris/HCl, 8 M Urea, 5% SDS, 1 mM EDTA, 0.02% bromophenol blue; pH 6.8). Samples were heated at 65 °C for 10 min, centrifuged for 30 s at 10,000× *g* and their proteins were separated on 12.5%-acrylamide gels using Tris-glycine running buffer (25 mM Tris Base, 200 mM Glycine, 0.05% SDS) and transferred to PVDF membranes (ROTH) using wet electro-transfer protocols. The following antibodies were used in this study: α-GFP (1:2500, mouse, 1181446001, Roche/Merck KGaA, Darmstadt, Germany), α-Tubulin (1:10,000, rabbit, Abcam, 184970, Cambridge, UK) and peroxidase-conjugated secondary antibodies against mouse (1:10,000, rabbit, A9044, Sigma/Merck KGaA, Darmstadt, Germany) or rabbit (1:10,000, goat, A0545, Sigma/Merck KGaA, Darmstadt, Germany). For chemiluminescence detection, luminol enhancer solution (ClarityTM Western ECL substrate; Bio-Rad, Solna, Sweden) and ChemiDoc XRS+ Imaging System (Bio-Rad, Solna, Sweden) were used, and images were processed using Image Lab ver.5.2.1 software (Bio-Rad, Solna, Sweden).

### 2.5. Fluorescence Microscopy

Cells were harvested at the indicated time points followed by resuspension in PBS. Dead cells were visualized by PI counterstaining. Epifluorescence microscopy pictures were taken with a ZEISS Axioplan2 microscope with 100×/1.4-oil objective by ZEISS Axio Cam MRm and the AxioVision 40 × 64 v4.9.1.0 software (Carl Zeiss Microscopy GmbH, Oberkochen, Germany). Confocal microscopy pictures were taken using an LSM800 Airyscan confocal microscope with a 63×/1.4-oil M27 objective using the ZEN blue software control (Carl Zeiss Microscopy GmbH, Oberkochen, Germany). Suitable filters were applied to visualize green fluorophores (GFP-tagged proteins) and red fluorophores (RedStar, mCherry-tagged proteins and PI staining). Micrographs were analyzed and processed using Fiji software [32]. Pictures within an experiment were captured and processed in the same way.

### 2.6. Analysis of mRNA Levels Using RT-qPCR

Approximately 2.5 × 10^8^ cells were harvested at indicated time points and total RNA was isolated using the Ribopure-Yeast™ kit (Thermo Fisher, AM1926). Quality of the extracted RNA was assessed using the Agilent 2100 bioanalyzer (G2939BA; Agilent, Santa Clara, CA, USA). Then, 2 μg of total RNA was reverse-transcribed following the SuperScript II Reverse protocol (Thermo Fisher, 18064014). Oligonucleotides used for RT-qPCR are listed in Supplemental Table S2. Primer efficiencies of 90–110% were confirmed, and RT-qPCR was carried out with the 2× KAPA SYBR^®^ Fast qPCR Master mix (SIGMA-ALDRICH, KK4600) in triplicates. *UBC6* or *RPN2* were used as housekeeping genes to calculate the relative gene expression levels with the comparative CT method (∆∆CT) [33].

### 2.7. Total Reflection X-ray Fluorescence (TXRF) Spectrometry

For whole cell multi-element analysis, 6 × 10^7^ cells were harvested by centrifugation (3 min, 3500× *g*), washed in 300 µL Milli-Q H_2_O, snap-frozen in liquid nitrogen and stored at −20 °C until further processing. Frozen cell pellets were resuspended in 100 µL 1% Triton X-100 at room temperature, mixed 1:1 with gallium standard solution (2 mg/L) and vortexed. Then, 10 µL of sample were transferred to TXRF quartz glass carriers and carefully dried on a hot plate. Data collection was carried out for 1000 s on an S2 PICOFOX (automatic) spectrometer (Bruker Nano GmbH, Karlsruhe, Germany) equipped with a molybdenum excitation source (50 kV/600 µA). Elements were assigned manually and spectra quantified in the PICOFOX^TM^ software (Bruker Nano GmbH, Karlsruhe, Germany). 

### 2.8. Quantification of Phosphate and Polyphosphate 

Acidic extracts to measure total P_i_ and polyP were obtained as previously described [34], with slight modifications. Briefly, washed yeast pellets of 1 × 10^7^ cells were frozen in liquid nitrogen and stored at –80 °C until further processing. Cell pellets were resuspended in 150 µL ice-cold 0.5-mM HClO_4_, incubated 30 min on ice, and centrifuged at 3000× *g* for 5 min at 4 °C. The supernatants were neutralized to pH 7–8 by the addition of (1 M KOH, 100 mM TrisHCl) and the resulting KClO_4_ was removed by centrifugation at 3000× *g* for 5 min at 4 °C. Long-chain polyP extraction was performed as described before [35]. PolyP levels were determined from the amount of P_i_ released upon treatment with an excess of purified recombinant exopolyphosphatase from *S. cerevisiae* (rPPX1), as described [34]. Briefly, samples of polyP extracts were incubated at 37 °C for 15 min with 60-mM Tris-HCl, pH 7.5, 6-mM MgCl_2_, and 3000–5000 units of purified rPPX1 in a final volume of 75 µL. The release of P_i_ was monitored according to the standard method of Lanzetta et al. [36], based on the colorimetric quantification of a green complex formed between malachite green, molybdate and free orthophosphate. 

### 2.9. Statistical Analysis

Data are presented as dot plots, line graphs or box plots. Dot plots show individual data points with lines depicting the mean and error bars showing standard error of the mean (sem). Line graphs are drawn with symbols depicting mean and error bars showing sem. Box plots show median (line) and mean (square) as well as whiskers, representing minima and maxima within the 1.5-interquartile range. The indicated sample size in the respective figure legends refers to biological replicates (number of independent cultures per condition). Statistical analysis was performed using GraphPad Prism (v8.0). T-test (unpaired) and analysis of variance (ANOVA) with Tukey’s post hoc tests were used for comparisons between two or multiple groups, respectively. Where appropriate, a two-way ANOVA was applied and corrected with Greenhouse–Geisser (when epsilon < 0.75). Outliers were identified using the ROUT method. Reported significance values are two-sided. Normal distribution of data was confirmed using Shapiro–Wilk’s test or judged based on visual inspection of *Q-Q* plots. Homogeneity of variance was tested using Brown Forsythe test. Welch’s test (Welch’s corrected ANOVA) with Dunnett T3-corrected post hoc comparisons were applied when variance was unequal. 

## 3. Results

### 3.1. Phosphate Restriction Induces Longevity

**Figure 1:**
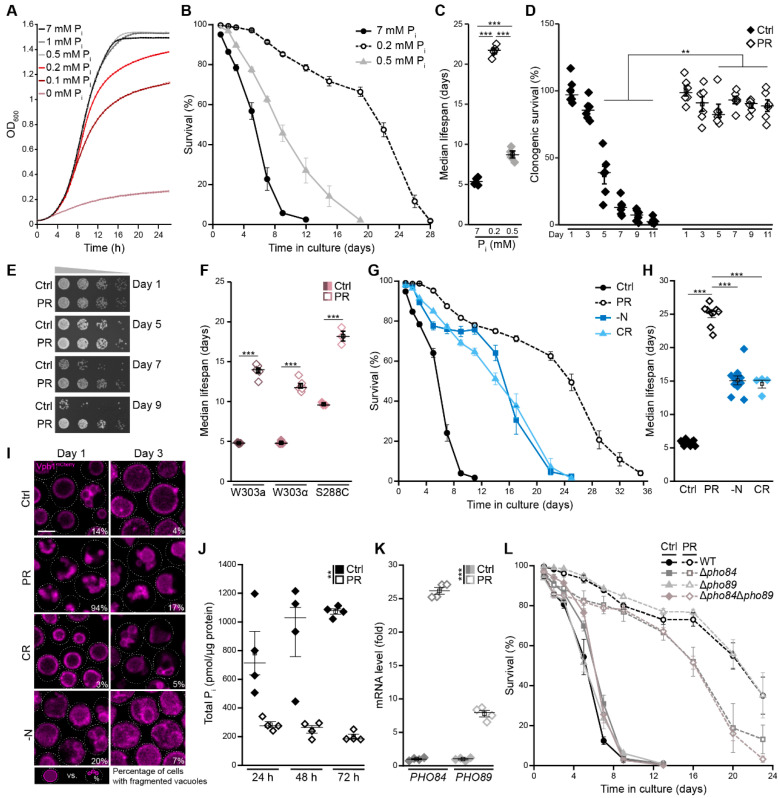
Phosphate restriction induces longevity. (**A**) Growth curves of wild type (WT; BY4742) cells on synthetic minimal glucose media with indicated phosphate (P_i_) concentrations. OD_600_ was recorded every 30 min using a BioscreenC microplate reader; *n* = 8. (**B**,**C**) Survival during chronological aging and (**B**) and corresponding median lifespan (**C**) of WT cells grown in media supplemented with 7 mM, 0.5 mM or 0.2 mM phosphate. Survival was determined by flow cytometric quantification of propidium iodide (PI) staining, indicating loss of membrane integrity and, thus, cell death; *n* = 4. (**D**) Clonogenic survival determined via survival plating and quantification of colony-forming units of WT cells grown on standard medium (Ctrl; 7 mM phosphate) or phosphate-restricted medium (PR; 0.2 mM phosphate). Survival was determined at indicated time points during chronological aging; *n* = 6. (**E**) Serial dilution spotting assays as a measure of regrowth capability of WT cells grown on Ctrl or PR media for indicated days. (**F**) Median lifespan of wild type yeast cells with different genetic backgrounds (W303a, W303α, and S288C) grown on Ctrl or PR media; *n* = 3–6; (**G**,**H**) Survival during chronological aging (**G**) and corresponding median lifespan (**H**) of WT (BY4742) cells grown under different nutrient conditions: nitrogen starvation (−N), caloric restriction (CR; 0.5% glucose), phosphate restriction (PR) (0.2 mM phosphate) and standard conditions (Ctrl). Survival was determined by flow cytometric quantification of PI staining; *n* ≥ 4. (**I**) Representative confocal micrographs of WT cells harboring endogenously mCherry-tagged Vph1 to visualize vacuoles grown for 1 day or 3 days under nutrient conditions shown in (**G**). Mean percentages of cells with multiple vacuoles are indicated (*n* = 4, with ≥42 cells per n and *n* = 4). Scale bar represents 3 µm. (**J**) Relative levels of total phosphate (P_i_) in WT cells grown for 24 h, 48 h and 72 h on standard or PR medium; *n* = 4. (**K**) The quantification of *PHO84* and *PHO89* mRNA levels via RT-qPCR in WT cells grown for 24 h on standard or PR medium. The comparative CT method (∆∆CT method) was used to calculate the relative gene expression using *UBC6* as housekeeping gene; *n* = 4. (**L**) Survival during chronological aging of WT, ∆*pho84,* ∆*pho89* and ∆*pho84*∆*pho89* cells on Ctrl or PR media. Survival was determined by flow cytometric quantification of PI staining; *n* = 4. For statistical analysis, one-way ANOVA followed by Tukey post hoc for multiple comparisons (**C**,**F**,**H**), two-way ANOVA (**D**,**J**) or Welch–ANOVA (**K**) were used. ** *p* < 0.01 and *** *p* < 0.001.

Upon cultivation on standard minimal media containing 2% glucose, cells exit cell cycle and enter quiescence as soon as glucose is exhausted [37]. To identify a setup in which cells enter stationary phase due to gradual depletion of phosphate instead of glucose but are still supplemented with sufficient phosphate to allow normal exponential growth, we monitored cellular growth across a range of phosphate concentrations. While almost no growth was visible in media completely lacking phosphate, already 0.5 mM was sufficient to enable growth comparable to standard glucose minimal media, containing 7 mM of phosphate. Supplementation with 0.2 mM phosphate yielded similar initial exponential growth but caused an early entry into stationary phase, indicating that cell-cycle exit was indeed induced by phosphate depletion (Figure 1A). The quantification of cellular viability in the stationary phase using flow-cytometric quantification of propidium iodide staining as a measure of cell death revealed that limiting phosphate to 0.5 mM mildly increased survival during aging, while a further reduction of phosphate availability to 0.2 mM resulted in a massive extension of lifespan (Figure 1B,C). Thus, we employed a nutritional regime with 0.2 mM phosphate—hereafter referred to as phosphate restriction (PR)—to assess the impact of gradual phosphate exhaustion and subsequent cell-cycle exit on cellular fitness during aging. The quantification of clonogenic survival, using classical plating assays, demonstrated that phosphate-restricted cells were able to regrow and form colonies, while cells in standard glucose conditions (Ctrl) gradually lost their colony-forming capacity (Figure 1D). Likewise, serial dilution spotting assays demonstrated a progressive loss of regrowth in standard conditions, while phosphate-exhausted cells retained their capability to regrow over time (Figure 1E). Further supporting the notion that PR results in cell-cycle exit and entry into quiescence, phosphate-restricted cells appeared to be almost exclusively unbudded (Supplemental Figure S1A) and no further increase in optical density over time was visible after day 2 (Supplemental Figure S1B). Collectively, these data suggest that PR results in longevity associated with efficient entry into and maintenance of quiescence. Acidification of the culture media has been shown to limit yeast survival in the stationary phase, and preventing media acidification, for instance via buffering to pH 6, efficiently extended yeast lifespan [38,39]. Thus, we tested whether a change in pH might contribute to PR-induced longevity. While phosphate-restricted cells acidified their surrounding slightly less than control cells (pH 2.8 versus pH 3.0), adjusting the pH of standard control media accordingly did not mimic the effects of PR (Supplemental Figure S1C,D). Hence, longevity of phosphate-restricted cells is not caused by decreased media acidification. To test whether PR would also result in longevity of yeast strains with different genetic background than BY4742, we monitored the lifespan of W303a, W303α and S288c in standard and phosphate-restricted conditions. Indeed, PR efficiently extended lifespan of all strains analyzed (Figure 1F and Supplemental Figure S1E–G).

Next, we directly compared the longevity induced by PR with the effects of two well-established dietary regimes known to extend lifespan: caloric restriction (CR), via limitation of glucose to 0.5%, and acute nitrogen starvation (−N). Though all nutrient-restrictive conditions resulted in an extension of lifespan, compared with standard glucose conditions (Ctrl), PR most efficiently induced longevity, resulting in an almost five-fold increase of median lifespan compared to a three-fold extension observed for the other dietary restrictions (Figure 1G,H). Cellular adaptation to nutrient starvation is accompanied by prominent organellar remodeling, which frequently includes an increase of the vacuolar volume-to-surface ratio. This not only amplifies vacuolar storage capacity but also serves to equip the vacuole with sufficient hydrolytic activity to ensure efficient breakdown of cargo delivered to the vacuole for subsequent recycling when nutrients are limited [40,41]. Visualization of vacuolar morphology using endogenously mCherry-tagged Vph1, a subunit of the V-ATPase, revealed that, in contrast to all other nutritional regimes, PR resulted in multiple small vacuoles in almost all cells in the early stationary phase, while, on later days, these numerous vacuoles fused to form one enlarged organelle (Figure 1I). As expected, restricting the availability of phosphate in the media resulted in a prominent reduction of total intracellular phosphate content (Figure 1J) and a transcriptional upregulation of the plasma membrane-localized high-affinity phosphate transporters Pho84 and Pho89 (Figure 1K), while gene expression of the low-affinity transporters Pho87 and Pho90 was reduced (Supplemental Figure S2A). Still, the lack of Pho84 and Pho89, alone or in combination, had only minor effects on PR-induced longevity (Figure 1L), though it mildly improved or reduced cellular survival shortly after entry into the stationary phase due to gradual depletion of glucose (Ctrl) or phosphate, respectively (Figure 1L and Supplemental Figure S2B). In sum, entry into the stationary phase, driven by the gradual exhaustion of phosphate instead of glucose, causes cellular changes that result in a prominent extension of lifespan.

### 3.2. Polyphosphate as a P_i_ Reservoir Is Dispensable for Longevity Induced by Phosphate Exhaustion

**Figure 2:**
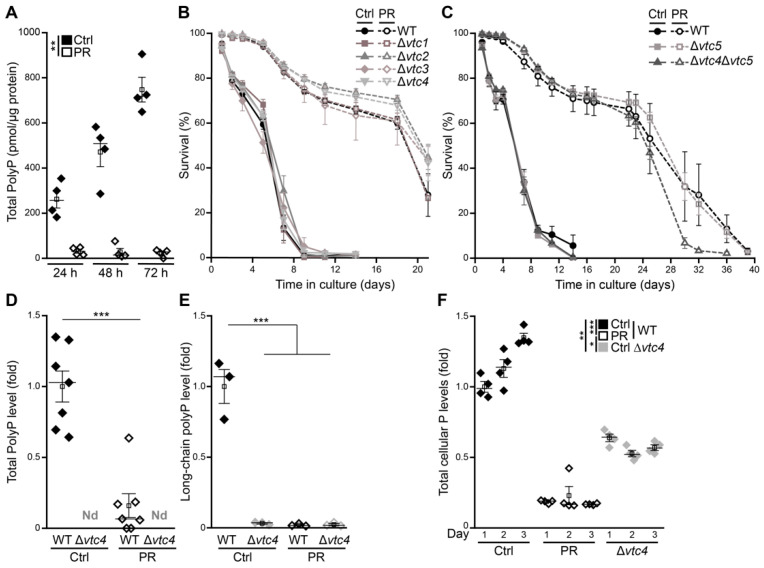
Polyphosphate as P_i_ reservoir is dispensable for longevity induced by gradual phosphate exhaustion. (**A**) Polyphosphate levels in WT cells grown for 24 h, 48 h and 72 h on standard (Ctrl) or phosphate-restriction (PR) media; *n* = 4. (**B**,**C**) Survival during chronological aging of WT and deletion mutants in genes coding for subunits of the vacuolar transporter chaperone (VTC) complex (∆*vtc1*, ∆*vtc2*, ∆*vtc3 and* ∆*vtc4*) (**B**) and WT and ∆*vtc5,* as well as the double-deletion mutant ∆*vtc4*∆*vtc5* (**C**) grown in Ctrl or PR media, determined by flow cytometric analysis of PI staining; *n* ≥ 3. (**D**) Total polyphosphate levels in WT cells and cells lacking Vtc4 grown for 24 h on Ctrl or PR medium (N.D., not detectable); *n* = 8 for WT and *n* = 3 for ∆*vtc4.* (**E**) Long-chain polyphosphate levels in samples described in (**D**); *n* = 3. (**F**) Total cellular phosphorus content (P) measured via total reflection X-ray fluorescence (TXRF) in WT and ∆*vtc4* cells grown in Ctrl or PR medium and harvested on days 1, 2 and 3. Values are presented as fold of WT Ctrl at day 1. *N* = 4. For statistical analysis, two-way ANOVA (**A**,**F**), unpaired-*T*-Test (**D**) and ordinary one-way ANOVA (**E**) were used. * *p* < 0.05, ** *p* < 0.01 and *** *p* < 0.001.

Cells store phosphate in the form of polyP, a linear polymer of tens to hundreds of P_i_ residues that preferentially accumulates in acidocalcisomes, organelles that are ubiquitous in nature [21]. In yeast cells, the vacuole represents the acidocalcisome-like compartment, accumulating polyP that can account for almost a quarter of their dry weight [42]. The concentration of cellular polyP fluctuates with the availability of phosphate, and polyP granules, formed under high phosphate conditions, serve as energy source and reservoir of P_i_ [20,21,43]. The quantification of total cellular polyP demonstrated that, upon glucose exhaustion, cells produced high levels of polyP throughout the first days of aging (Figure 2A). In cells entering stationary phase due to gradual phosphate exhaustion, polyP was completely absent (Figure 2A). To test whether polyP would be necessary to allow cellular adaptation to reduced phosphate availability, we monitored survival of cells defective in polyP synthesis. However, deletion of genes coding for subunits of the heteromeric vacuolar transporter chaperone (VTC) complex, which connects synthesis of polyP to its transport into the vacuole, affected neither viability during aging under control conditions nor longevity induced by PR (Figure 2B,C). The absence of both short-chain as well as long-chain polyP in a *VTC4* deletion mutant demonstrated that indeed synthesis and storage of polyP was completely absent in cells lacking a functional VTC complex (Figure 2D,E). PolyP deficiency translated into a 50% reduction of total cellular phosphorus levels in ∆*vtc4* cells grown in control conditions, as determined using total reflection X-ray fluorescence spectrometry (Figure 2F). In sum, this shows that polyP as phosphate reservoir and buffering system for vacuolar pools of cations is completely dispensable for cellular survival in a post-mitotic state and PR still efficiently induces longevity of cells that lack the capability to store phosphate.

### 3.3. Phosphate Exhaustion Results in a Strong Induction of Autophagy 

**Figure 3:**
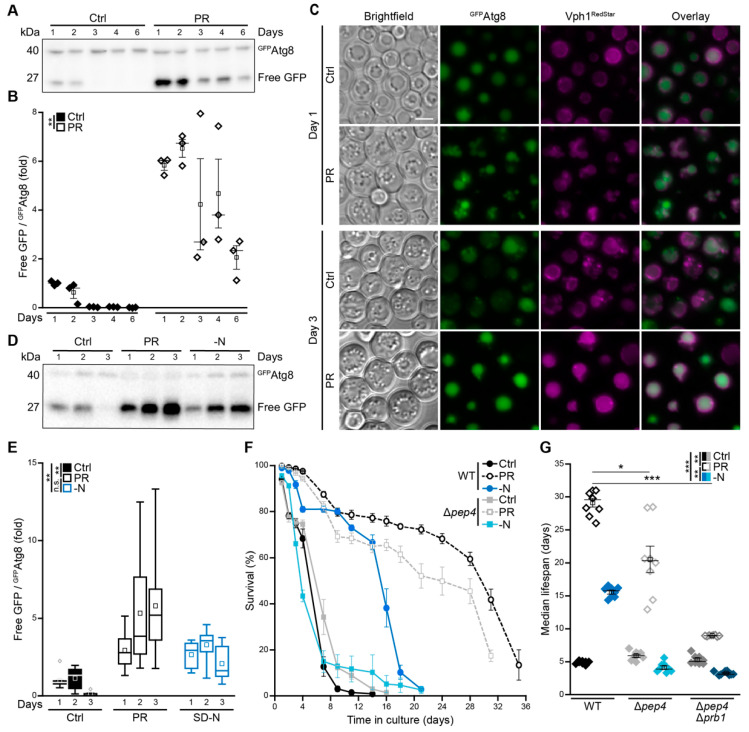
Phosphate exhaustion results in a strong induction of autophagy. (**A**,**B**) Immunoblot analysis (**A**) and corresponding densitometric quantification (**B**) of ^GFP^Atg8 processing in protein extracts from WT cells harboring endogenously GFP-tagged Atg8 grown in standard (Ctrl) or phosphate-restriction (PR) media. Blots were probed with antibodies directed against GFP, and the ratio free GFP/^GFP^Atg8, indicative of autophagic flux, is depicted; *n* = 3. (**C**) Microscopic analysis of WT cells endogenously expressing ^GFP^Atg8, as well as Vph1^RedStar^, to visualize vacuoles grown for 1 or 3 days on Ctrl or PR media. Scale bar represents 3 µm. (**D**,**E**) Immunoblot analysis (**D**) and corresponding densitometric quantification (**E**) of ^GFP^Atg8 processing in protein extracts from WT cells harboring GFP-tagged Atg8 subjected to phosphate restriction (PR), nitrogen starvation (−N) or standard conditions (Ctrl). Blots were probed with antibodies directed against GFP, and the ratio free GFP/^GFP^Atg8, indicative of autophagic flux, is depicted; *n* = 8. (**F**) Survival during chronological aging of WT and ∆*pep4* cells grown on Ctrl, PR or −N medium. Cell death was determined via flow cytometric analysis of PI staining; *n* ≥ 4. (**G**) Median lifespan of cells shown in (**F**) including ∆*pep4*∆*prb1* cells; *n* = 8. For statistical analysis, two-way ANOVA (**B**,**E**) and Welch–ANOVA (**G**) were used. * *p* < 0.05, ** *p* < 0.01 and *** *p* < 0.001; *ns* not significant.

Across species barriers, CR has been shown to extend cellular and organismal lifespan at least in part via an induction of autophagy, the main cellular bulk-degradation process [3,5]. To assess how gradual phosphate exhaustion impacts on autophagy, we employed yeast cells expressing an endogenously GFP-tagged variant of Atg8 under the control of its native promoter [44]. Atg8 localizes to autophagosomal membranes and is targeted to the vacuole as a consequence of autophagosome fusion with the vacuole. Thus, the ^GFP^Atg8 chimera represents a functional marker for autophagosome formation and for vacuolar autophagic breakdown, which generates free GFP as an estimate of the completed autophagic process. Immunoblot analyses demonstrated that PR resulted in a rapid and massive activation of autophagy as indicated by increased GFP liberation (Figure 3A,B). In line, fluorescence microscopy revealed a clear accumulation of cells with GFP-positive vacuoles, prognostic of functional autophagosomal clearance (Figure 3C). During aging in standard conditions, autophagic activity dropped within the first three days, while PR efficiently induced autophagic flux throughout the complete time span monitored (Figure 3A–C). A direct comparison with acute nitrogen starvation, a regime that prominently upregulates autophagy [15,45,46,47], revealed that growing cells into phosphate depletion activated autophagy in a comparable or even more potent manner (Figure 3D,E). As efficient recycling of autophagic cargo upon delivery to the vacuole requires hydrolytic breakdown, we next evaluated whether Pep4, the main vacuolar protease in yeast and counterpart of mammalian cathepsin D [31,48,49], would be essential to achieve full lifespan extension via PR. Interestingly, though acute nitrogen starvation as well as gradual phosphate exhaustion resulted in a prominent induction of autophagy, the requirement of vacuolar proteolytic capacity for full lifespan extension differed substantially (Figure 3F,G). In line with previous reports showing that Pep4 is strictly required for survival and vacuolar autophagic degradation under nitrogen starvation [47,48,50], we observed a complete loss of nitrogen starvation-induced prolongation of cellular lifespan. In contrast, the absence of Pep4 only slightly reduced PR-induced longevity (Figure 3F,G), and only the simultaneous lack of Pep4 and proteinase B (Prb1) prominently impaired PR-induced extension of lifespan (Figure 3G and Supplemental Figure S3A,B). Thus, despite a clear activation of autophagy upon gradual phosphate exhaustion, the proteolytic activity of Pep4 was not required for lifespan extension.

### 3.4. Autophagy Supports Long-Term Survival of Phosphate-Limited Cells

**Figure 4:**
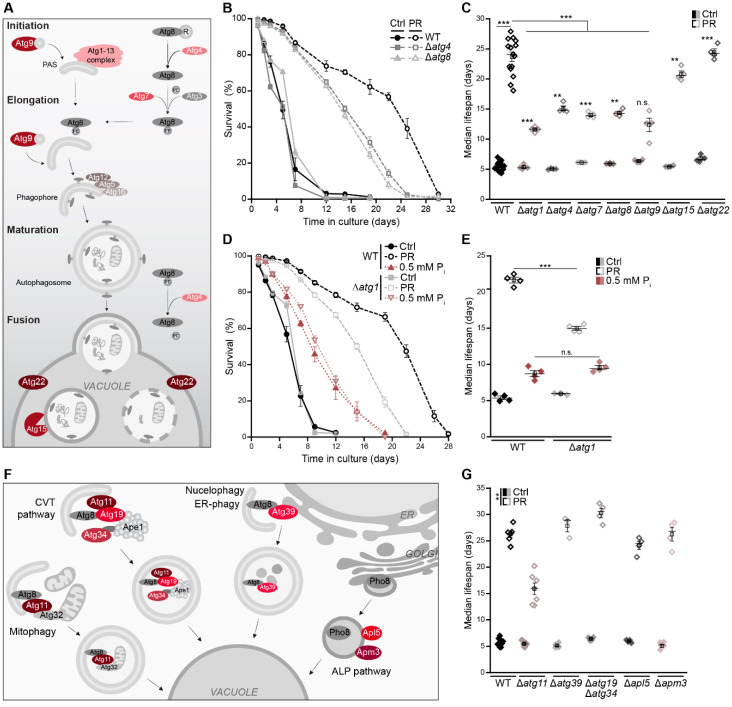
Autophagy supports long-term survival of phosphate-limited cells. (**A**) Schematic overview of key players involved in macroautophagy. (**B**) Survival during chronological aging, determined via flow cytometric quantification of PI staining, of WT, ∆*atg4* and ∆*atg8* cells grown on standard (Ctrl) or phosphate-restriction (PR) media; *n* = 4. (**C**) Median lifespan of WT cells and mutants lacking core autophagy genes *(ATG1, ATG4, ATG7, ATG8, ATG9, ATG15* and *ATG22*) grown on Ctrl and PR media; *n* ≥ 4. (**D**,**E**) Survival during chronological aging (**D**) and corresponding median lifespan (**E**) of WT and ∆*atg1* cells grown in media containing standard phosphate concentrations (7 mM; Ctrl), 0.5 mM or 0.2 mM phosphate (PR); *n* = 4. (**F**) Schematic overview of selective autophagy and the ALP pathway. (**G**) Median lifespan of WT cells and mutants lacking genes involved in selective autophagy and the ALP pathway, as depicted in (**F**), grown on Ctrl or PR media; *n* ≥ 3. For statistical analysis, Welch–ANOVA with Dunnett T3 post hoc (**C**,**F**) and one-Way ANOVA with Tukey post hoc (**E**) were used. ** *p* < 0.01 and *** *p* < 0.001.

To test whether the induction of autophagy represents a prerequisite for PR-induced longevity, we monitored the lifespan of several autophagy-deficient mutants upon entry into stationary phase driven by glucose versus phosphate exhaustion. The deletion of genes coding for components of the core autophagic machinery involved in autophagy initiation and autophagosome formation, including Atg1, Atg4, Atg7, Atg8 and Atg9 (Figure 4A), reduced but did not prevent PR-induced longevity (Figure 4B,C). During early days of aging, these deletion mutants responded to phosphate starvation just as wild-type cells. However, long-term survival was decreased, indicating that cells need autophagic turnover late during phosphate starvation to sustain survival (Figure 4B,C and Supplemental Figure S4A,B). The vacuolar lipase Atg15, absolutely required for nitrogen starvation-induced lifespan extension [51], as well as the vacuolar permease Atg22, involved in amino acid recycling [52], and Pho23, a transcriptional activator of Atg9 [53], were dispensable for PR-induced longevity (Figure 4C and Figure S4B–D). Interestingly, the intermediate lifespan prolongation achieved via culturing on 0.5 mM phosphate was completely independent of autophagy (Figure 4D,E). 

To further assess the contribution of selective autophagy to PR-induced longevity, we analyzed a set of yeast mutants defective in mitophagy and other forms of selective autophagy (∆*atg11*), nucleophagy (∆*atg39*) and the anabolic cytoplasm-to-vacuole targeting (CVT) pathway (∆*atg19*∆*atg34*), which shuttles distinct hydrolytic enzymes to the vacuole [54]. In addition, we monitored the lifespan of cells defective in the alkaline phosphatase (ALP) pathway (∆*apl5* and ∆*apm3*), mediating Golgi-to-vacuole transport of Pho8, a phosphatase upregulated upon phosphate starvation [55,56] (Figure 4F). None of these pathways were essential for longevity induced by gradual phosphate exhaustion (Figure 4G and Supplemental Figure S4E,F). Still, the lack of Atg11 compromised full lifespan extension comparable to the loss of core components of the autophagic machinery. Whether this contribution of Atg11 to longevity upon gradual phosphate exhaustion reflects a dedicated function of Atg11 as scaffold for a specific form of selective autophagy remains to be explored, as Atg11 coordinates numerous forms of selective autophagic turnover and regulates steps ranging from cargo selection to autophagosome maturation [57]. Notably, a previous study suggested that Atg11 but not its cargo recognition domain contributes to mild autophagy induction following acute phosphate starvation [58]. 

Thus, despite a massive induction of autophagy shortly after phosphate is running out, cellular survival during the first week of post-mitotic aging does not require a functional autophagy machinery. However, full lifespan extension upon PR depends on the presence of the core machinery of autophagy. This suggests that an induction of autophagy early during stationary phase without immediate effects on survival primes the cell for persisting starvation, supporting long-term cellular fitness.

### 3.5. Autophagy and the Multivesicular Body Pathway Work in Parallel to Sustain Survival upon Phosphate Exhaustion

**Figure 5:**
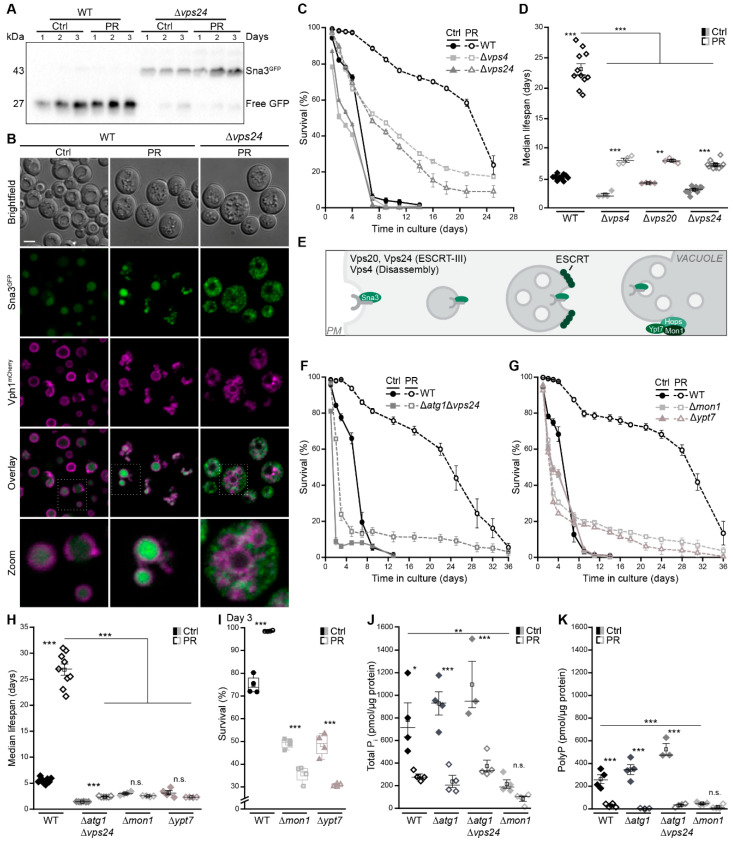
Autophagy and the multivesicular body pathway work in parallel to sustain survival upon phosphate exhaustion. (**A**) Immunoblot of WT and ∆*vps24* cells harboring endogenously GFP-tagged Sna3 grown in standard (Ctrl) or phosphate-restriction (PR) media. Blots were probed with an antibody against GFP to assess GFP liberation. (**B**) Confocal microscopy of WT and ∆*vps4* cells harboring endogenously GFP-tagged Sna3 and mCherry-tagged Vph1. Images of ∆*vps4* cells were post-processed differently to visualize Sna3^GFP^. Scale bar represents 3 µm. (**C**) Survival during chronological aging, determined via flow cytometric quantification of PI staining, of WT, ∆*vps4* and ∆*vps24* cells grown on Ctrl or PR media; *n* = 4. (**D**) Median lifespan of WT, ∆*vps4*, ∆*vps20* and ∆*vps24* cells. (**E**) Schematic showing the multivesicular body (MVB) pathway and key players of vacuole fusion. (**F**–**H**) Survival during chronological aging of WT and ∆*atg1*∆*vps4* cells (**F**) and of WT, ∆*mon1* and ∆*ypt7* cells (**G**) grown on Ctrl or PR media, as well as corresponding median lifespan (**H**); *n* > 4. (**I**) Survival of WT, ∆*mon1* and ∆*ypt7* cells on day 3 of the aging shown in (**G**). (**J**,**K**) Total phosphate (**J**) and polyphosphate (**K**) levels in WT, ∆*atg1*, ∆*atg1*∆*vps4* and ∆*mon1* cells grown for 24 h on Ctrl or PR media; *n* = 4, except ∆*atg1*∆*vps4 n* = 3. For statistical analysis, Welch-ANOVA with Dunnett T3 post hoc test (**D**,**H**) and one-Way ANOVA with Tukey post hoc test (**I**–**K**) was used. * *p* < 0.05, ** *p* < 0.01 and *** *p* < 0.001; *ns* not significant.

The MVB pathway delivers plasma membrane cargo to the vacuole and is critical for cellular survival upon glucose or nitrogen depletion [18,59,60]. Thus, we assessed the contribution of the MVB pathway and its molecular sorting machinery, the endosomal sorting complex required for transport (ESCRT), to PR-induced lifespan extension (Figure 5A–E). Monitoring the turnover of endogenously GFP-tagged Sna3, a cargo of the MVB pathway, revealed an activation of the MVB pathway upon phosphate deprivation (Figure 5A,B). Vacuolar GFP liberation from Sna3^GFP^ was increased (Figure 5A), and Sna3^GFP^ accumulated in the vacuole (Figure 5B). This was strictly ESCRT-dependent, as Sna3^GFP^ shuttling to the vacuole as well as cleavage within the vacuole were absent in cells lacking Vps24 (Figure 5A,B). Viability of cells defective in the MVB pathway, due to lack of either Vps24, Vps20 or Vps4, dropped rapidly upon phosphate exhaustion, and lifespan prolongation was strongly compromised, though not completely abolished (Figure 5C,D and Supplemental Figure S5A). Thus, MVB pathway activity is critical to sustain viability upon phosphate exhaustion, in particular during the early phase of aging, while autophagy is required for long-term survival. Simultaneous inactivation of autophagy and the MVB pathway in cells devoid of Atg1 and Vps24 completely abrogated PR-induced lifespan prolongation (Figure 5F). Autophagosomes and MVBs fuse with the vacuolar membrane to release an autophagic body or intraluminal vesicles, respectively, into the vacuolar lumen for hydrolytic degradation and subsequent recycling of macromolecules. Thus, a functional vacuolar fusion machinery is required for the final turnover (Figure 5E). In line with a complete loss of PR-induced longevity upon simultaneous disruption of autophagy and the MVB pathway, the lack of either Mon1 or Ypt7, both essential for vesicle tethering and subsequent fusion with the vacuole, precluded lifespan extension (Figure 5G,H). While cells lacking Mon1 or Ypt7 died early after glucose exhaustion, this death was even accelerated upon phosphate restriction (Figure 5I), suggesting an increased need for functional fusion of vesicles with the vacuole. Quantification of phosphate levels revealed that selectively the lack of Mon1 resulted in a severe drop of total phosphate and a complete absence of polyP when cells entered stationary phase, both upon glucose or phosphate exhaustion, while the inactivation of autophagy and the MVB pathway did not impact phosphate levels (Figure 5J,K). 

Notably, we observed that the loss of functional vacuolar fusion, as well as the simultaneous inactivation of autophagy and the MVB pathway, resulted in a small number of escapers that maintained viability during the complete aging (Figure 5F,G). This hints towards alternative mechanisms that compensate for the absence of these pathways in individual cells and contribute to the full spectrum of cellular responses to phosphate exhaustion.

### 3.6. The Cyclin-Dependent Kinase Pho85 Is Critical for Phosphate Exhaustion-Induced Autophagy

**Figure 6:**
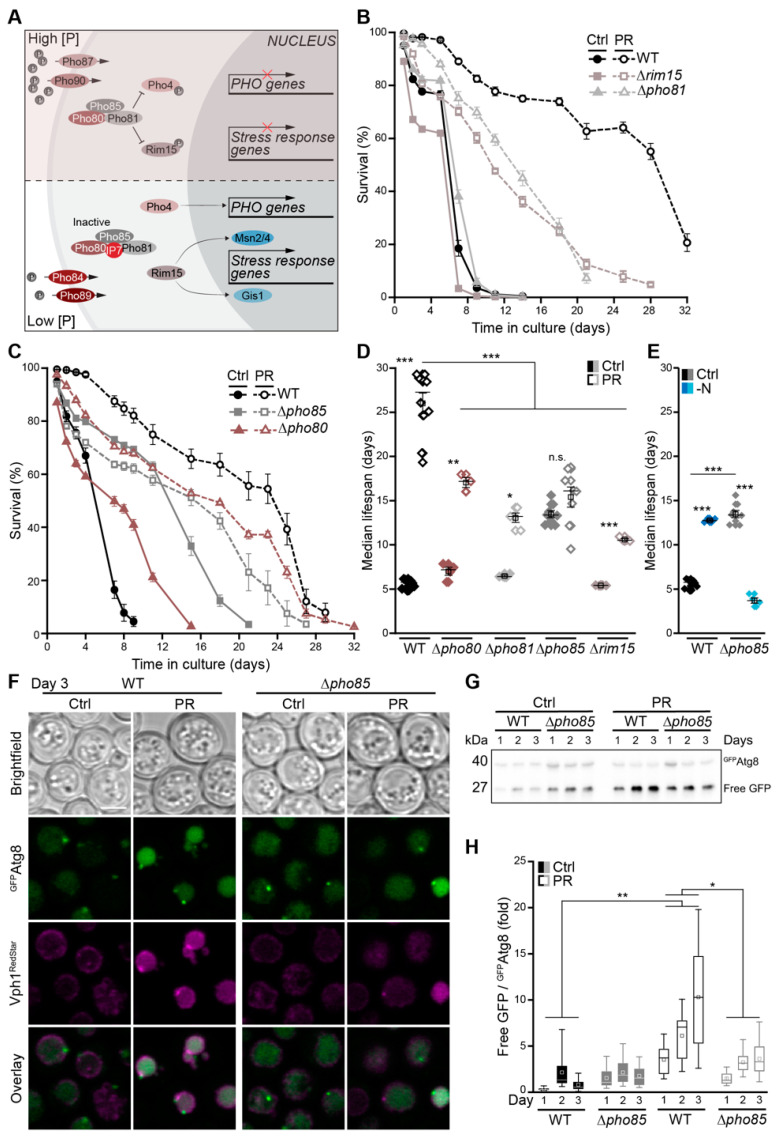
The cyclin-dependent kinase Pho85 is critical for phosphate exhaustion-induced autophagy. (**A**) Schematic representation of the yeast phosphate signaling (PHO) pathway. (**B**–**D**) Survival during chronological aging, determined via flow cytometric quantification of PI staining, of WT, ∆*rim15* and ∆*pho81* cells (**B**), and of WT, ∆*pho80* and ∆*pho85* cells (**C**) grown on Ctrl or PR media; *n* = 4. Corresponding median lifespan is shown in (**D**). (**E**) Median lifespan of WT and ∆*pho85* cells grown on Ctrl or nitrogen starvation (−N) medium; *n* = 4. (**F**) Confocal micrographs of WT and ∆*pho85* cells harboring ^GFP^Atg8 and Vph1^RedStar^ (as vacuolar marker) at day 3 of aging on Ctrl or PR media. Scale bar represents 3 µm. (**G**,**H**) Representative immunoblot (**G**) and corresponding densitometric quantification (**H**) of protein extracts from WT and ∆*pho85* cells harboring endogenously GFP-tagged Atg8 grown on Ctrl or PR medium. Blots were decorated with an antibody against GFP, and the ratio free GFP/^GFP^Atg8 was plotted; *n* = 8. For statistical analysis, Welch–ANOVA with Dunnett T3 post hoc test (**D**) or a two-way ANOVA (**H**) was used. * *p* < 0.05, ** *p* < 0.01 and *** *p* < 0.001.

Several evolutionarily conserved nutrient-sensing kinases are involved in the regulation of autophagy upon starvation, including the protein kinase A (PKA) and Sch9 as parts of the central glucose signaling pathways, as well as the TORC1 complex sensing nitrogen and amino acids [3,7,61,62]. In response to TORC1 inhibition, the stress-responsive cyclin-dependent kinase Pho85, an orthologue of mammalian CDK5, has been shown to positively or negatively regulate autophagy, depending on its respective cyclin [63]. Upon phosphate starvation, repression of the Pho85–Pho80 complex by the inhibitor Pho81 and increased levels of myo-D-inositol heptakisphosphate (IP7) result in the activation of Rim15 and Pho4, which, in turn, induced genetic reprogramming to enable phosphate assimilation via the PHO pathway, efficient entry into quiescence and stress resistance (Figure 6A) [64,65]. In line, the lack of Rim15-induced stress-response genes prominently reduced PR-induced longevity (Figure 6B). Consistently, cells devoid of Pho81 to repress the Pho85–Pho80 complex lost viability early in stationary phase upon phosphate exhaustion (Figure 6B). Deletion of the genes coding for Pho85 and its cyclin Pho80 resulted in lifespan prolongation, per se, when cell-cycle exit was triggered by glucose exhaustion in the control medium. When entry into quiescence was driven by phosphate exhaustion, the lack of this complex reduced lifespan extension (Figure 6C,D). Interestingly, Pho85 was strictly required for longevity induced by acute nitrogen starvation (Figure 6E and Supplemental Figure S5B). Using a ^GFP^Atg8 chimera to monitor autophagy during the first 3 days of glucose versus phosphate exhaustion, we found that the lack of Pho85 resulted in a moderate activation of autophagy early after glucose depletion. In contrast, the massive induction of autophagy upon PR in wild type cells was reduced in cells lacking Pho85 (Figure 6F–H). Thus, while Pho85 functions as negative regulator of autophagy when cells adapt to glucose limitation, this kinase is essential to induce autophagy in phosphate-depleted cells.

## 4. Discussion

Phosphate is an essential macronutrient that serves as crucial building block for an array of macromolecules. Cells harbor systems to sense phosphate availability, to establish and maintain appropriate phosphate levels and to store a surplus in form of polyP. We show that a gradual depletion of phosphate, driving entry into stationary phase, results in a prominent extension of yeast lifespan. Within hours after phosphate exhaustion, cells are depleted of polyP, indicating that they use this phosphate reservoir very efficiently for cellular adaptation. Still, complete inactivation of polyP synthesis and storage via genetic ablation of the VTC complex did not affect PR-induced entry into quiescence and longevity, suggesting that the levels of phosphate necessary to sustain essential cellular processes can be retrieved from alternative sources. Our results reveal that cells respond to phosphate limitation with an induction of two major catabolic pathways: autophagy, facilitating the degradation of cytoplasmic material, and the MVB pathway, which functions in the turnover of plasma membrane material. Interestingly, genetic inactivation of the MVB pathway prevented pro-survival effects of PR already during early days of aging, while cells defective in autophagy still responded efficiently early on but displayed a prominent drop in survival at late stages of aging. Thus, a sequential and coordinated action of the MVB pathway and autophagy is crucial for PR-induced longevity. Accordingly, preventing fusion of MVBs and autophagosomes with the vacuole via genetic inactivation of the vacuolar tethering and fusion machinery resulted in a complete abrogation of lifespan extension by phosphate limitation. Interestingly, the CVT pathway, which equips the vacuole with distinct hydrolases, was completely dispensable for PR-induced longevity. Moreover, inactivating the major vacuolar protease Pep4 had only minor effects on viability upon phosphate exhaustion. Whereas the loss of Pep4 resulted in immediate cell death upon nitrogen starvation, PR still efficiently extended the lifespan of these protease-deficient cells. Only the simultaneous lack of Pep4 and Prb1 resulted in a reduction (but not complete inhibition) of longevity upon PR, suggesting that the cellular requirement for specific vacuolar proteolytic capacities differs depending on the respective nutrient scarcity.

Cells sense and adapt to phosphate limitation via Pho85, a multifunctional kinase that mediates the cell cycle, the transition into quiescence and responses to environmental stimuli in concert with multiple cyclins [42,66,67]. Upon nitrogen starvation or rapamycin treatment, leading to an inhibition of TORC1, the lack of Pho85 has been shown to activate autophagy [63,68]. This function of Pho85 as negative regulator of rapamycin-induced autophagy has been linked to specific cyclins, including Clg1, Pcl1, and Pho80, which in complex with Pho85 mediate the destabilization of the cell-cycle inhibitor Sic1 [63]. Here, we establish Pho85 and its cyclin Pho80 as positive regulators of autophagy upon gradual phosphate depletion. Genetic ablation of Pho85 prevented the PR-induced activation of autophagy and impaired concomitant lifespan extension. In contrast, when cells entered stationary phase driven by gradual glucose depletion, the lack of Pho85 resulted in lifespan prolongation and a modest induction of autophagy, indicating that this kinase has opposing functions in autophagy regulation in response to nutrient deprivation. 

The Pho85–Pho80 inhibitor Pho81, which associates with the complex independently of phosphate levels but inhibits Pho85 kinase activity only under phosphate limitation, was required for full lifespan extension. This likely is due to the inability of cells devoid of Pho81 to fully activate the stress response transcription factor Rim15, essential for efficient entry into quiescence [65,69]. Accordingly, the loss of Rim15 itself reduced PR-induced longevity. Thus, while Pho85 inactivation via its inhibitor as well as subsequent activation of Rim15-mediated transcriptional reprogramming is essential for full lifespan extension via PR, complete inactivation of the Pho85–Pho80 complex still compromised lifespan under phosphate depletion, likely due to insufficient induction of autophagy. On the other hand, when cells entered stationary phase due to glucose exhaustion, still supplied with sufficient phosphate, inactivation of Pho85–Pho80, genetically mimicking low phosphate, resulted in lifespan extension and subtle autophagy induction. In response to general nutrient depletion, the activities of nutrient-sensing kinases such as the Pho85–Pho80 complex, PKA signaling and the TORC1 pathway integrate to adapt cell-cycle exit and cellular functions to the respective nutritional stress [70,71], and depending on the limiting macronutrient driving entry into stationary phase, Pho85 functions as positive or negative regulator of autophagy and lifespan. 

In mammals, phosphate and aging seem tightly linked, and high serum phosphate levels are correlated with decreased lifespan [72,73]. High dietary phosphate intake has been associated with increased mortality in a human study [74], and excess serum phosphate levels are linked to age-associated pathologies, in particular chronic kidney disease [72,75]. Here, dietary phosphate restriction as well as phosphate binders represent a common therapeutic approach to counteract hyperphosphatemia. Moreover, genetic inactivation of key regulators of phosphate homeostasis, including fibroblast growth factor-23 (FGF23) as well as its co-receptor, klotho, not only result in overaccumulation of phosphate but also prominently shorten the lifespan of mice [72,76,77]. Counteracting hyperphosphatemia via reduction of phosphate intake suppressed premature aging, supporting the notion that excess phosphate indeed accelerates aging. Similar to caloric restriction, a low phosphate diet has been shown to affect insulin-responsive genes and oxidative stress responses [72,78], fundamental processes that have been connected to the aging process in a wide range of species. In *Drosophila*, the restriction of phosphate intake has been shown to extend lifespan, though the cellular and molecular mechanisms involved remain elusive [79,80]. Although the key regulators of yeast phosphate homeostasis lack obvious counterparts in higher eukaryotes, yeast lifespan extension by phosphate restriction involves autophagy and the MVB pathway as fundamental and evolutionarily highly conserved processes. Hence, the use of yeast to unravel molecular mechanisms controlling the metabolic changes and cellular adaptations in response to phosphate restriction will further our understanding of the tight connection between phosphate and cellular aging. 

## Data Availability

The data used to support the findings of this study are included in the article.

## Supplementary Materials

The following are available online at https://www.mdpi.com/article/10.3390/cells10113161/s1, Figure S1: Phosphate exhaustion results in efficient entry into quiescence and longevity in different genetic backgrounds; Figure S2: Gradual phosphate exhaustion leads to transcriptional downregulation of low-affinity phosphate transporters; Figure S3: Simultaneous lack of the vacuolar proteases Pep4 and Prb1 impairs phosphate restriction-induced longevity; Figure S4: The core machinery of autophagy is required for full lifespan extension via phosphate restriction; Figure S5: The MVB pathway and Pho85 are critical for survival upon nutrient limitation; Table S1: Yeast strains used in this study; Table S2: Oligonucleotides used for gene deletion, chromosomal tagging and RT-qPCR.
