# Supplementary material for: Phosphate Restriction Promotes Longevity via Activation of Autophagy and the Multivesicular Body Pathway
Source: Cells. 2021 Nov 13;10(11):3161. doi: 10.3390/cells10113161 (PMC8620443; doi:10.3390/cells10113161)
Supplement: Supplementary file 1 [file cells-10-03161-s001.zip › cells-1396255-supplementary.pdf]

Supplemental Material

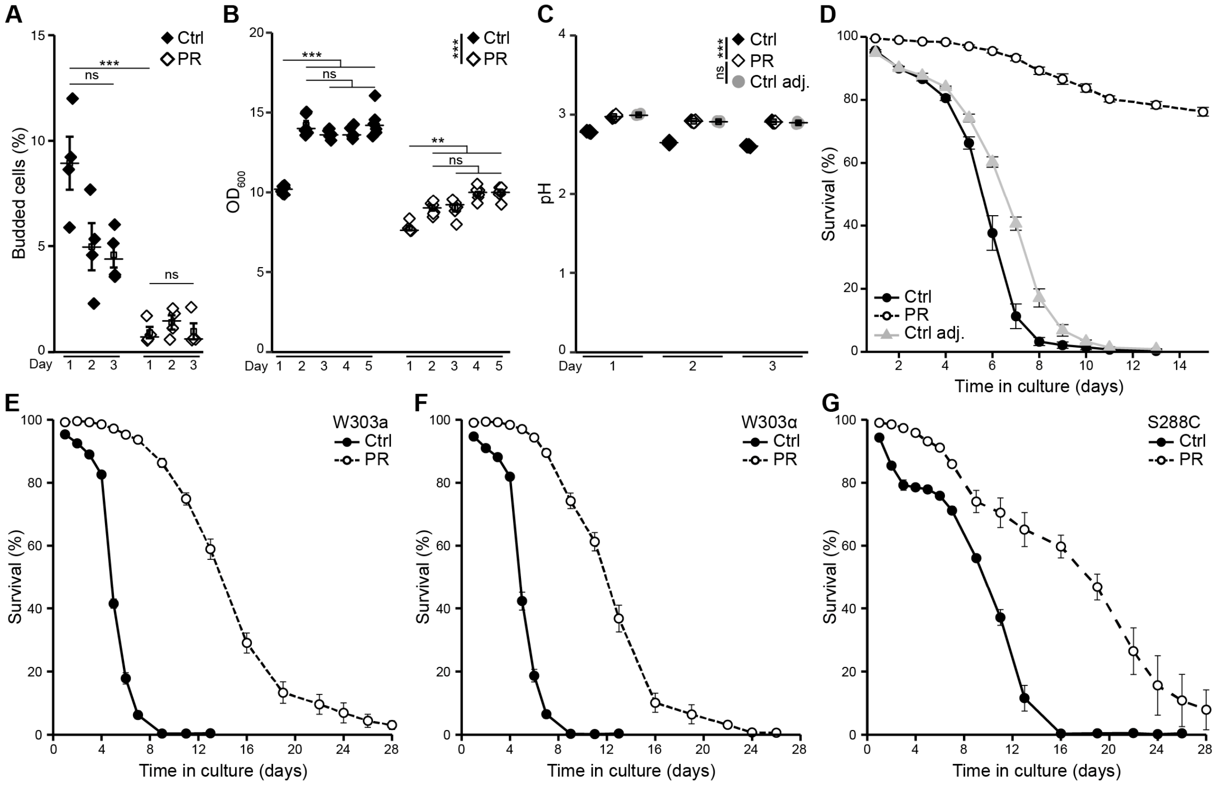

Figure S1. Phosphate exhaustion results in efficient entry into quiescence and longevity in different genetic backgrounds**.** (**A**) Quantification of budded WT cells cultured on standard medium (Ctrl) and phosphate-restricted medium (PR) for indicated days. Cells were manually counted, and percentage of budded cells is shown; *n* = 4, with at least 100 cells per experiment and condition. (**B**) Optical density (OD_{600}) of WT cells cultured in Ctrl and PR media, determined at indicated time points during aging; *n* = 6. (**C**) Determination of culture media pH after WT cells have been cultured for indicated days on standard medium (Ctrl), phosphate-restricted medium (PR) and standard medium with pH adjustment to the media pH of PR cultures (Ctrl adj.). (**D**) Survival during chronological aging of WT cells cultured as described in (**C**). Survival was determined by flow cytometric analysis of PI staining; *n* = 5. (**E-G**) Survival of the yeast wild type strains W303a (**E**), W303α (**F**), and S288c (**G**) during chronological aging on Ctrl and PR medium. Survival was determined by flow cytometric analysis of PI staining at indicated days during aging; n=3-6. For statistical analysis, two-way ANOVA (**A, B, C**) was used. **$p < 0.01$ and ***$p < 0.001$; *ns* not significant.

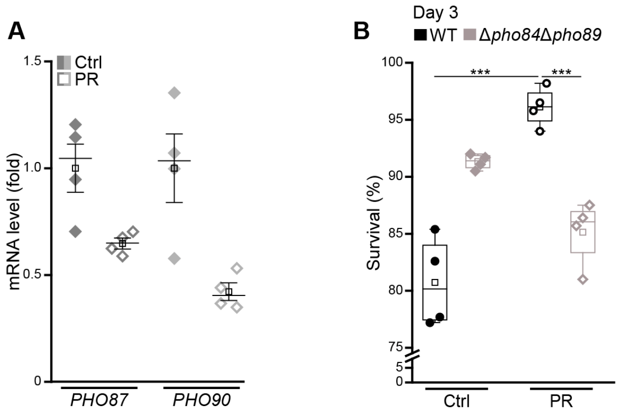

Figure S2. Gradual phosphate exhaustion leads to transcriptional downregulation of low-affinity phosphate transporters. **(A)** Quantification of *PHO87* and *PHO90* mRNA levels via RT-qPCR in WT cells grown for 24 h on standard (Ctrl) or phosphate restriction (PR) medium. Comparative CT method (ΔΔCT method) was used to calculate the relative gene expression using *UBC6* as housekeeping gene; $n = 4$. (**B**) Survival of WT cells and cells lacking both high-affinity phosphate transporters (Δ*pho84*Δ*pho89*) on day 3 of chronological aging on Ctrl or PR medium, corresponding to the survival curve shown in Fig. 1L; $n = 4$. For statistical analysis, one-way ANOVA followed by Tukey post hoc for multiple comparisons (**B**) was used. ***$p < 0.001$.

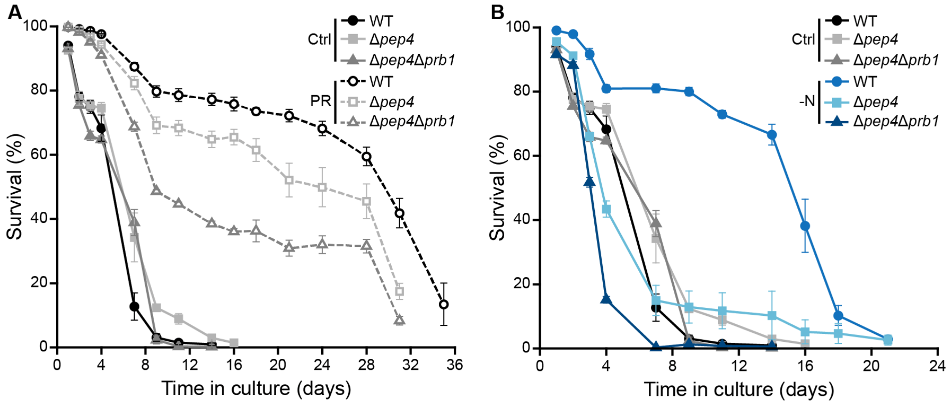

Figure S3. Simultaneous lack of the vacuolar proteases Pep4 and Prb1 impairs phosphate restriction-induced longevity. Survival during chronological aging, determined via flow cytometric quantification of PI staining, of WT, Δ*pep4* and Δ*pep4*Δ*prb1* cells grown under different nutrient conditions: phosphate restriction (PR), nitrogen starvation (−N) and standard conditions (Ctrl). For clarity, survival curves are shown in two separate graphs for phosphate restriction (**A**) and nitrogen starvation (**B**); $n \geq 4$.

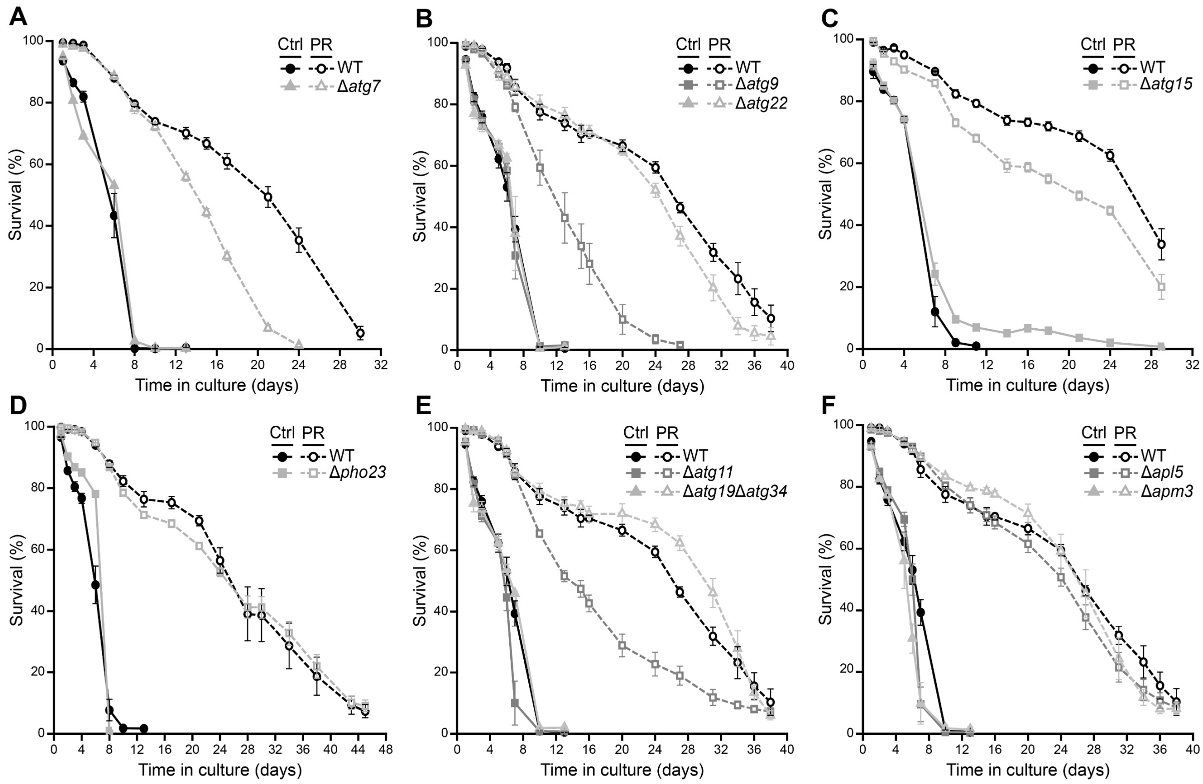

Figure S4. The core machinery of autophagy is required for full lifespan extension via phosphate restriction. Survival during chronological aging, determined via flow cytometric quantification of PI staining, of WT, Δ*atg7* (**A**), Δ*atg9*, Δ*atg22* (**B**), Δ*atg15* (**C**), Δ*pho23* (**D**), Δ*atg11*, Δ*atg19*Δ*atg34* (**E**), and Δ*apl5* and Δ*apm3* (**F**) cells grown on standard (Ctrl) or phosphate restriction (PR) medium; $n \geq 4$.

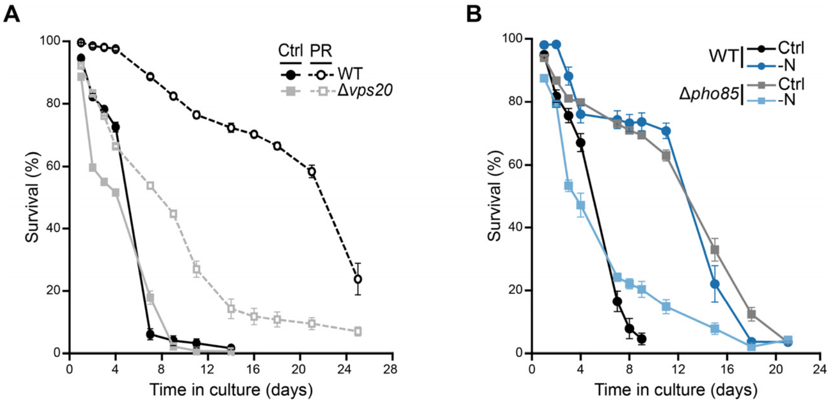

Figure S5. The MVB pathway and Pho85 are critical for survival upon nutrient limitation. Survival during chronological aging, determined via flow cytometric quantification of PI staining, of WT and Δ*vps20* cells grown on standard (Ctrl) or phosphate-restricted (PR) medium; $n \geq 4$ (**A**), as well as of WT and *Δpho85* cells grown on Ctrl or -N media; $n = 4$ (**B**).

Table S1. Yeast strains used in this study.

Strain	Genotype	Source
BY4742	MATα, *his3* 1, *leu2* 0, *lys2* 0, *ura3* 0	Euroscarf
W303a	MATa, *ade2-1, ura3-1, his3-11, trp1-1, leu2-3, leu2-112, can1-100*	
W303α	MATα, *ade2-1, ura3-1, his3-11, trp1-1, leu2-3, leu2-112, can1-100*	
S288C	*MATα, SUC2, gal2, mal2, mel, flo1, flo8-1, hap1, ho, bio1, bio6*	
BY4742 Vph1^{mCherry}	BY4742 *VPH1*-mCherry-natNT2	This study
pho84	BY4742 *pho84* ::hphNT1	This study
pho89	BY4742 *pho89* ::hphNT1	This study
pho84 pho89	BY4742 *pho84* ::hphNT1, *pho89* ::natNT2	This study
pho87	BY4742 *pho87* ::hphNT1	Euroscarf
pho90	BY4742 *pho90* ::hphNT1	Euroscarf
vtc1	BY4742 *vtc1* ::hphNT1	This study
vtc2	BY4742 *vtc2* ::hphNT1	This study
vtc3	BY4742 *vtc3* ::hphNT1	This study
vtc4	BY4742 *vtc4* ::hphNT1	This study
vtc5	BY4742 *vtc5* ::natNT2	This study
vtc4 vtc5	BY4742 *vtc4* ::hphNT1,*vtc5* ::natNT2	This study
BY4742 ^{GFP}Atg8	BY4742 p*ATG8* ::natNT2-pATG8-EGFP	This study
BY4742 ^{GFP}Atg8 Vph1^{Redstar}	BY4742 p*ATG8* ::natNT2-pATG8-EGFP, *VPH1*-Red-star-kanMX	This study
pep4	BY4742 *pep4* ::hphNT1	This study
pep4 prb1	BY4742 *pep4* ::hphNT1, *prb1* ::kanMX	This study
atg1	BY4742 *atg1* ::hphNT1	This study
atg4	BY4742 *atg4* ::hphNT1	This study
atg7	BY4742 *atg7* ::hphNT1	This study
atg8	BY4742 *atg8* ::hphNT1	This study
atg9	BY4742 *atg9* ::hphNT1	This study
atg11	BY4742 *atg11* ::hphNT1	This study
atg15	BY4742 *atg15* ::hphNT1	This study
atg22	BY4742 *atg22* ::hphNT1	This study
atg19 atg34	BY4742 *atg19* ::hphNT1, *atg34* ::kanMX	This study
atg39	BY4742 *atg39* ::hphNT1	This study
apl5	BY4742 *apl5* ::hphNT1	This study
apm3	BY4742 *apm3* ::hphNT1	This study
pho23	BY4742 *pho23* ::hphNT1	This study
BY4742 Sna3^{GFP}	BY4742 *SNA3*-EGFP-kanMX	This study
BY4742 Sna3^{GFP} Vph1^{mCherry}	BY4742 *SNA3*-EGFP-KanMX, *VPH1*-mCherry-natNT2	This study
vps24	BY4742 *vps24* ::hphNT1	This study
vps24 Sna3^{GFP}	BY4742 *vps24* ::hphNT1, *SNA3*-yeGFP-kanMX	This study
vps24 Sna3^{GFP} Vph1^{mCherry}	BY4742 *vps24* ::hphNT1, *SNA3*-yeGFP-kanMX, *VPH1*-mCherry-natNT2	This study
vps4	BY4742 *vps4* ::hphNT1	This study
vps20	BY4742 *vps20* ::hphNT1	This study
atg1 vps24	BY4742 *atg1* :: kanMX, *vps24* ::kanMX	Ralf Braun
mon1	BY4742 *mon1* ::natNT2	This study
ypt7	BY4742 *ypt7* ::hphNT1	This study
rim15	BY4742 *rim15* ::hphNT1	This study

pho80	BY4742 *pho80* ::hphNT1	This study
pho81	BY4742 *pho81* ::hphNT1	This study
pho85	BY4742 *pho85* ::hphNT1	This study
BY4742 ^{GFP}Atg8 *pho85*	BY4742 pATG8 ::natNT2-pATG8-EGFP, *pho85* ::hphNT1	This study
BY4742 ^{GFP}Atg8 *pho85* Vph1^{Redstar}	BY4742 pATG8 ::natNT2-pATG8-EGFP, *pho85* ::hphNT1, *VPH1*-Redstar-kanMX	This study

Table S2. Oligonucleotides used for gene deletion, chromosomal tagging and RT-qPCR.

Modification	Oligonucleotides	PCR template
	**Deletion and tagging of genes**	
C-terminal tagging of *VPH1*	5′-GAAGTACTTAAATGTTTCGCTTTTTTTAAAAGTCCTCAAAAT TTAATCGATGAATTCGAGCTCG-3′<br>5′-GACATGGAAGTCGCTGTTGCTAGTGCAAGCTCTTCCGCTTC AAGCCGTACGCTGCAGGTCGAC-3′	pYM42-mCherry-natNT2 [1]<br>pYM38 [2]
Control PCR *VPH1* tagging	5′-CGAGCTCGAATTCATCGAT-3′<br>5′-GTATTCGAGGCCAATACTTG-3′	
Deletion of *PHO84*	5′-CGTAGATCACCAGGGCACACAACAAACAAAACTCCACGAA TACAATCCAA ATG CGTACGCTGCAGGTCGAC-3′<br>5′-GTATTATTTGTTCTAGTTTACAAGTTTTAGTGCATCTTTG AG-GCTTTTAATCGATGAATTCGAGCTCG-3′	pFA6a-hphNT1 [2]
Control PCR *PHO84* deletion	5′-CGTTCCTCTCACTGCCGCAC-3′<br>5′-GTCGACCTGCAGCGTACG-3′	
Deletion of *PHO89*	5′-AATACTGGAGCGTATCTAATCGAATCAATATAAACAAAGATT AAGCAAAA ATG CGTACGCTGCAGGTCGAC-3′<br>5′-TACCTAATGTTGTAAAACTG TATTCATAACCGAAACACATT ATGATTAATCGATGAATTCGAGCTCG-3′	pFA6a-hphNT1 [2]<br>pFA6a-natNT2 [2]
Control PCR *PHO89* deletion	5′-GAATATACCGTACAAAGGAG-3′<br>5′-GTCGACCTGCAGCGTACG-3′	
Deletion of *VTC1*	5′-CGATTTACTACATTATCGAATACGATTAAACACTACGCC AGAT-TTCCACAATATGCGTACGCTGCAGGTCGAC-3′<br>5′-GAAAATACAGTTTGTGCGTAACCCACGCTTACGATATT GGAATTACAATTTCAATCGATGAATTCGAGCTCG-3′	pFA6a-hphNT1 [2]
Control PCR *VTC1* deletion	5′-GGACCAGTTTTATAAGAAGGT-3′<br>5′-GTCGACCTGCAGCGTACG-3′	
Deletion of *VTC2*	5′-GTAGAAAGAACGACTACACCTCAACATAACGACACTT TTTT-GACATG CGTACGCTGCAGGTCGAC-3′<br>5′-GCTACACATTACAAACATAAAAACACATGGTCTCAGTAGA TA-GAGTACATATTCTAATCGATGAATTCGAGCTCG-3′	pFA6a-hphNT1 [2]
Control PCR *VTC2* deletion	5′-CTGGAAGGTATTTTCATCAG-3′<br>5′-GTCGACCTGCAGCGTACG-3′	
Deletion of *VTC3*	5′-GGCTATTAGAGCGAACAGCAGAATTTGTCCTTGGTTTTCA GAG-TTTGAAA ATGCGTACGCTGCAGGTCGAC-3′<br>5′-GATAGTAACTGGTACTTGTGTAATATATGTGTATATAAAAA AT-ATACATGTTCTTAATCGATGAATTCGAGCTCG-3′	pFA6a-hphNT1 [2]
Control PCR *VTC3* deletion	5′-GGATGTTAATGAGTAACTTG-3′<br>5′-GTCGACCTGCAGCGTACG-3′	
Deletion of *VTC4*	5′-GCTAACAATCAAATCGGCCAATAAAAGAGCATAACAAGGC AG-GAACAGCTATGCGTACGCTGCAGGTCGAC-3′	pFA6a-hphNT1 [2]

	5′-CTAATATGATTATTACTTAATTATACAGTAAAAAAAACA CGCTGTGTATTCAATCGATGAATTCGAGCTCG-3′	
Control PCR *VTC4* deletion	5′-TGTAAAGCCAGTACCACAAG-3′<br>5′-GTCGACCTGCAGCGTACG-3′	
Deletion of *VTC*5	5′-TATCTCCATCTCAGCCCTAGGTAAGGATAGATACGAAGGG TTGTACAATCATGCGTACGCTGCAGGTCGAC-3′<br>5′-CATATTTTATATAGACTGTACACATGAAAAACTTTTATC GTCCAAAATAATTAATCGATGAATTCGAGCTCG-3′	pFA6a-natNT2 [2]
Control PCR *VTC5* deletion	5′-CATTACACCCGTACGTAATC-3′<br>5′-GTCGACCTGCAGCGTACG-3′	
Deletion of *ATG8*	5′-CTAATAATTGTAAAGTTGAGAAAATCATAATAAAATAATTACTA GAGACATGCGTACGCTGCAGGTCGAC-3′<br>5′-CTATAATTTCGATTTTAGATGTTAACGCTTCATTTCTTTTC ATA-TAAAAGACTAATCGATGAATTCGAGCTCG-3′	pFA6a-hphNT1 [2]
N-terminal tagging of *ATG8*	5′-CTAATAATTGTAAAGTTGAGAAAATCATAATAAAATAATTAC TAGAGACATGCGTACGCTGCAGGTCGAC-3′<br>5′-GACTCCGCCTTCCTTTTTTCAAATGGATATTCAGACTTAA ATGTAGACTTCATCGATGAATTCTCTGTCG-3′	pYM–pATG8 [3]
Control PCR *ATG8* deletion	5′-CTAACTGTCTCCACCGATAATG-3′<br>5′-GTCGACCTGCAGCGTACG-3′	
Control PCR *ATG8* tagging	5′-GAACAATAGATGGCTAATGAGTCC-3′<br>5′-GGTTATGGTGTTCAATGTTTTGC-3′	
Deletion of *PEP4*	5′-GTGACCTAGT ATTTAATCCA AATAAAATTC AAACAAAAAC CAAAACTAAC ATG CGTACGCTGCAGGTCGAC-3′<br>5′-CTAGATGGCAGAAAAGGATAGGGCGGAGAAGTAAGAAAAGT TTAGCTCA ATCGATGAATTCGAGCTCG-3′	pFA6a-hphNT1 [2]
Control PCR *PEP4* deletion	5′-CATAATCCTGCTTGATGTGG-3′<br>5′-GTCGACCTGCAGCGTACG-3′	
Deletion of *PRB1*	5′-ATCGCCAATAAAAAAACAAACTAAACCTAATTCTAACAA GCAAAGCAGCTGAAGCTTCGTACGC-3′<br>5′-CGCCTAAGGAAAGAAAAAGAAAAAAAAAAGCAGCTGAA ATTTTTCTAAATGAAGAAGCATAGGCCACTAGTGGATCTG-3′	pUG6 [4]
Control PCR *PRB1* tagging	5′-GAGATGGACGAGAAACAGGG-3′<br>5′-GCGTACGAAGCTTCAGCTG-3′	
Deletion of *ATG1*	5′-ACCCCATATTTTCAAATCTCTTTTACAACACCAGACGAGA AATTAAGAAAATGCGTACGCTGCAGGTCGAC-3′<br>5′-ATATAGCAGGTCATTTGTACTTAATAAGAAAACCATATTATG CATCACTTAATCGATGAATTCGAGCTCG-3′	pFA6a-hphNT1 [2]
Control PCR *ATG1* deletion	5′-GTAATGTAAGGAAAACCCAC-3′<br>5′-GTCGACCTGCAGCGTACG-3′ ′	
Deletion of *ATG4*	5′-GTTAGTAGATGAAGAATGGACGACTTCTTATCACGTATAGG AG-TGATATACATGCGTACGCTGCAGGTCGAC-3′<br>5′-GAATATATTAAAACAAGTATATATGCTTATGAACTAGTGAA TTCCTTACACTAATCGATGAATTCGAGCTCG-3′	pFA6a-hphNT1 [2]
Control PCR *ATG4* deletion	5′-CGCCTACATCATCAAGATCAC-3′<br>5′-GTCGACCTGCAGCGTACG-3′	
Deletion of *ATG7*	5′-ATAACTAAAGTTCATTATATTTCAACAAATATAAGATAAT CAA-GAATAAAATGCGTACGCTGCAGGTCGAC-3′<br>5′-CGGAAAGTGGCACCACAATATGTACCAATGCTATTATATGC AAAATATTAATCGATGAATTCGAGCTCG-3′	pFA6a-hphNT1 [2]

Control PCR *ATG7* deletion	5′-TGGAACGTGCAGAAAAGGAC -3′<br>5′-GTCGACCTGCAGCGTACG-3′	
Deletion of *ATG9*	5′-GACATTTAAGAACAGCCTGAAATATCAAAATCACGGAATTAT TAGGTTATGCGTACGCTGCAGGTCGAC-3′<br>5′-CAGTTATATATATAGTTATATTGGATGATGTACACGACAC AG-TCTGCCTTAATCGATGAATTCGAGCTCG-3′	pFA6a-hphNT1 [2]
Control PCR *ATG9* deletion	5′-CGAGCTCGAATTCATCGAT-3′<br>5′-TGCAAAGATGGCTCATATTG-3′	
Deletion of *ATG11*	5′-GTGTACTGTTGTTGTTCGGAAAGTACTTCTTTTATTTTCT TTTATA-CATCATGCGTACGCTGCAGGTCGAC-3′<br>5′-GATACATAATTAAAATCTTGTCATTTGTGACAAACGTTTAGCA CTGTTCAATCGATGAATTCGAGCTCG-3′	pFA6a-hphNT1 [2]
Control PCR *ATG11* deletion	5′-GCTAGCATTCCTATATATCC-3′<br>5′-GTCGACCTGCAGCGTACG-3′	
Deletion of *ATG15*	5′-AACTGATCTAGGCATTACAATTAAAGGAAACAAGGGAAAT ATTCTATTGAATGCGTACGCTGCAGGTCGAC-3′<br>5′-CGCATAGGCCCTAAAACAACACTAGGGTCATAATAGATGT ATGGGTCTTAATCGATGAATTCGAGCTCG-3′	pFA6a-hphNT1 [2]
Control PCR *ATG15* deletion	5′-TATAATAAGCATAACATCGG-3′<br>5′-GTCGACCTGCAGCGTACG-3′	
Deletion of *ATG22*	5′-GCCCTTATAAGCAATAGTTTGCTCATAACATATTCTCTA CATT-AGATATG CGTACGCTGCAGGTCGAC-3′<br>5′-CCTTATATATATACGTAAGCAAAGTTTTATGTAACAAA AAAAAAAAAAGAACTAATCGATGAATTCGAGCTCG-3′	pFA6a-hphNT1 [2]
Control PCR *ATG22* deletion	5′-GTTAATCATCCTCCAGCAAAGC-3′<br>5′-GTCGACCTGCAGCGTACG-3′	
Deletion of *ATG19*	5′-GGTAATTGCGGCGGCACTTGCTTCAGTAACGCCCAA AGGAGAG-TTCTGGTAAATGCGTACGCTGCAGGTCGAC-3′<br>5′-GTGTATGTGAAAAGGTACTCATTGCTGTATAAAAA TAGAGTTT-GACCTAATCGATGAATTCGAGCTCG-3′	pFA6a-hphNT1 [2]
Control PCR *ATG19* deletion	5′-CGCGTTCCATTTGAGAAGC-3′<br>5′-GTCGACCTGCAGCGTACG-3′	
Deletion of *ATG34*	5′-GTTAAATAAGTACTATAGCCAAAGAAACTGGAAGAATA TAAAAAAGCATGCATAGGCCACTAGTGGATCTG-3′<br>5′-GAAACTAGTTCCTATAGGTTGAGTGTCTATCAAA AATTTACGGA-GACGCGCAGCTGAAGCTTCGTACGC-3′	pUG6 [4]
Control PCR *ATG34* deletion	5′-GGTCGTATCCTTTCGACAGTG-3′<br>5′-GCGTACGAAGCTTCAGCTG-3′	
Deletion of *ATG39*	5′-GCAGTGACGATAATAGAGACTAGTAAAACAGTCGAGTTGT CGGACCTAAAATGCGTACGCTGCAGGTCGAC-3′<br>5′-CGTTTTTTTTTTCTTTTGTTAATTTCATTCTTCATGCTGGGTT TTGGATGATCTAATCGATGAATTCGAGCTCG-3′	pFA6a-hphNT1 [2]
Control PCR *ATG39* deletion	5′-GCTGCATATTTGCTTTCGCCG-3′<br>5′-GTCGACCTGCAGCGTACG-3′	
Deletion of *APL5*	5′-CTGAAGTGGG AAAGGCAGTTGCCACAACCAGAAGAGCATA ACATATTGTCATGCGTACGCTGCAGGTCGAC-3′<br>5′-TACGTACGTAATCGCTGTCATTGAGAATTCACGACTTCG ATTTTTTTATCGTTAATCGATGAATTCGAGCTCG-3′	pFA6a-hphNT1 [2]
Control PCR *APL5* deletion	5′-CGAAGCTTTTCATCCTAACGAAC-3′<br>5′-GTCGACCTGCAGCGTACG-3′	
Deletion of *APM3*	5′-GACTTGGGCA ACAACAGAGGCTGTAAACCTTACCAACCCA AC-CAAAATAG ATGCGTACGCTGCAGGTCGAC-3′	pFA6a-hphNT1 [2]

	5′-TCATTATATTCTATTTAGTTTCGCATGGAATTTCAAGTA CACATA-<br>TAACTAATCGATGAATTCGAGCTCG-3′	
Control PCR *APM3* deletion	5′-CAGTAATTGCATTCACAACCTGTG-3′<br>5′- GTCGACCTGCAGCGTACG-3′	
Deletion of *PHO23*	5′-CTTGCAGTATCACAAACAGCAACCACGTTCATAGTCTGAA<br>ATACGTACAAATGCGTACGCTGCAGGTCGAC-3′<br>5′-GTATTTAGTAGATATTTCCTTAAGGCGATATTCGCCCGAG AGC-<br>TATTTCAATCGATGAATTCGAGCTCG-3′	pFA6a-hphNT1<br>[2]
Control PCR *PHO23* deletion	5′-ATCAGGCGCCTTAGACCGCT-3′<br>5′-GTCGACCTGCAGCGTACG-3′	
C-terminal tagging of *SNA3*	5′-CGATGTGCCCTTGATGGACAACAAACAACAGCTCTCTTCC<br>GGCCGTACTCGTACGCTGCAGGTCGAC-3′<br>5′-CTCTATGTGTTATTTAGAACTACGGTGATAAAGAGCTCGT<br>TCCGATCACTAATCGATGAATTCGAGCTCG-3′	pYM27<br>[2]
Control PCR *SNA3* tagging	5′-GTCGACCTGCAGCGTACG-3′<br>5′-GCACGTGATG AAGGCTCGTT-3′	
Deletion of *VPS24*	5′-ACCTTTAGTAGTTTGGGGGGCAGTTTTCTGGGCAATACAAAG<br>TTTA CTTTTGATGCGTACGCTGCAGGTCGAC-3′<br>5′-CATTATTTATTCACTTATTTATTTATTTTCTTTGTACAGTCACAG<br>TAACACTCTAATCGATGAATTCGAGCTCG-3′	pFA6a-hphNT1<br>[2]
Control PCR *VPS24* deletion	5′-CGCAACTCCCACTCCCAATA-3′<br>5′-GTCGACCTGCAGCGTACG-3′	
Deletion of *VPS4*	5′-ATGGAAGACAAAAATAAAGCAGCATAGAGTGCCTATAGTAGA<br>TGGG GTACAAATGCGTACGCTGCAGGTCGAC-3′<br>5′-TTTTTATTTTCATGTACACAAGAAATCTACATTAGCACGTTAA<br>TCAATTGACTAATCGATGAATTCGAGCTCG-3′	pFA6a-hphNT1<br>[2]
Control PCR *VPS4* deletion	5′-CAGTCGCGCCAACGACCAGT-3′<br>5′-GTCGACCTGCAGCGTACG-3′	
Deletion of *VPS20*	5′-ATAGTACAGACTGCTGAATTAACTCCACTT GGTGCTTTTG TATA-<br>TATCGA ATG CGTACGCTGCAGGTCGAC-3′<br>5′-CTATTTACATTCCCTTTATTTTTAATTTTGAAGCTACATACAGA<br>CATTCA ATCGATGAATTCGAGCTCG-3′	pFA6a-hphNT1<br>[2]
Control PCR *VPS20* deletion	5′-GAGGGGTTCTCTAACGCTGT-3′<br>5′-GTCGACCTGCAGCGTACG-3′	
Deletion of *MON1*	5′-GAATAAAAAAGTCTATCAAAGTACACAAACGTAGAATCAGTA<br>CATCGGAACTATGCGTACGCTGCAGGTCGAC-3′<br>5′-GATATATTTACAAATTAAAGGAAAAAATAATAAAATAACCTCC<br>CTGTCACAAGTTAATCGATGAATTCGAGCTCG-3′	pFA6a-natNT2<br>[2]
Control PCR *MON1* deletion	5′-CGAAAAGCAGTTCAAGCTCAC-3′<br>5′-GTCGACCTGCAGCGTACG-3′	
Deletion of *YPT7*	5′-CCACTTCTTATCCATATAGA AACCCCTTCTGTATCAATTC<br>AAATTAAGTGATGCGTACGCTGCAGGTCGAC-3′<br>5′-AAGGATACGCTATAAAGGATTACATAATAGAAGATACAAT<br>TAAGTAGTACAGCTCAATCGATGAATTCGAGCTCG-3′	pFA6a-hphNT1<br>[2]
Control PCR *YPT7* deletion	5′-TATATACGGGCAGCTCCGTA-3′<br>5′-GTCGACCTGCAGCGTACG-3′	
Deletion of *RIM15*	5′-TTTCTCTTGCCTCATTTGATAGAATAGATAAGCCCAGTAGA<br>GGAAGACAGATGCGTACGCTGCAGGTCGAC-3′<br>5′-TTTCAAGCAAAGTTTTTATTCAGTTATTTTTTTAATTATCT<br>TTATCTTAAAATTTATCAATCGATGAATTCGAGCTCG-3′	pFA6a-hphNT1<br>[2]

Control PCR *RIM15* deletion	5′-CTAATATATAGTGAATGTAGGGAGC-3′<br>5′-GTCGACCTGCAGCGTACG-3′	
Deletion of *PHO80*	5′-CAAATTTAAGTCTGCAAGCTATCATAAGACGAGGATATCC TTT-GGAGACTCATAGAAATCATGCGTACGCTGCAGGTCGAC-3′<br>5′-TGGAAAGTTTTAATTTTGCTCAATCATGATTGCTTTCA TAA-TACCCCACGAAAAATCATTA ATCGATGAATTCGAGCTCG-3′	pFA6a-hphNT1 [2]
Control PCR *PHO80* deletion	5′-TTAACGAATTATGGTATTCT-3′<br>5′-GTCGACCTGCAGCGTACG-3′	
Deletion of *PHO81*	5′-CTTTTTGAGAGAATAACCCTTTGGAGGCAACATAGATAGA TAAACGTGCAATGCGTACGCTGCAGGTCGAC-3′<br>5′-AAAAGTTTTTCCTAAATAATGTATAAGATTTCAAA AC-TACATATTACAGAACTTTAATCGATGAATTCGAGCTCG-3′	pFA6a-hphNT1 [2]
Control PCR *PHO81* deletion	5′-CTAATTGCATCAGCCTATCTTTC-3′<br>5′-GTCGACCTGCAGCGTACG-3′	
Deletion of *PHO85*	5′-CAGATAAGGGATATATAGCGCGGCAAACTGGGCAAAC TTGAG-CAATACCAATGCGTACGCTGCAGGTCGAC-3′<br>5′-ATTATTATCATTATATATACATGGCTACGGTTTTTCGCT GAC-GGGCTGCGTTAATCGATGAATTCGAGCTCG-3′	pFA6a-hphNT1 [2]
Control PCR *PHO85* deletion	5′-GTCATTGTTGAGAGAAAGGAG-3′<br>5′-GTCGACCTGCAGCGTACG-3′	
	**RT-qPCR**	
PHO84	5′-AGCTTGGGGTCAAATCTCCG-3′<br>5′-TCAGCACCAGAGTTTGCGTA-3′	
PHO89	5′-CCAGTGCTTTGATTGGCTCG-3′<br>5′-GCGCCAATTGTACCACCAAC-3′	
PHO87	5′-ATGGGGTTGGCTTCATCTGG-3′<br>5′-AGCGCCTACAGTTAGCCATC-3′	
PHO90	5′-GTGGGTACTTTCGTCTCGCA-3′<br>5′-TAGGAGCAGCTTTGGGGTTG-3′	
UBC6	5′-GGACGTTTCAAGCCCAACAC-3′<br>5′-TGAGACAGACCAGCCAGGAT-3′	

References

1. Tosal-Castano, S.; Peselj, C.; Kohler, V.; Habernig, L.; Berglund, L.L.; Ebrahimi, M.; Vögtle, F.-N.; Höög, J.; Andréasson, C.; Büttner, S. Snd3 Controls Nucleus-Vacuole Junctions in Response to Glucose Signaling. *Cell Rep.* **2021**, *34*, 108637, doi:10.1016/j.celrep.2020.108637.
2. Janke, C.; Magiera, M.M.; Rathfelder, N.; Taxis, C.; Reber, S.; Maekawa, H.; Moreno-Borchart, A.; Doenges, G.; Schwob, E.; Schiebel, E.; et al. A Versatile Toolbox for PCR-Based Tagging of Yeast Genes: New Fluorescent Proteins, More Markers and Promoter Substitution Cassettes. *Yeast* **2004**, *21*, 947–962, doi:10.1002/yea.1142.
3. Eisenberg, T.; Schroeder, S.; Andryushkova, A.; Pendl, T.; Küttner, V.; Bhukel, A.; Mariño, G.; Pietrocola, F.; Harger, A.; Zimmermann, A.; et al. Nucleocytosolic Depletion of the Energy Metabolite Acetyl-Coenzyme A Stimulates Autophagy and Prolongs Lifespan. *Cell Metab.* **2014**, *19*, 431–444, doi:10.1016/j.cmet.2014.02.010.
4. Gueldener, U.; Heinisch, J.; Koehler, G.J.; Voss, D.; Hegemann, J.H. A Second Set of LoxP Marker Cassettes for Cre-Mediated Multiple Gene Knockouts in Budding Yeast. *Nucleic Acids Res.* **2002**, *30*, e23.
